# Role of Artificial Intelligence in Cleft Lip and/or Cleft Palate in Diagnosis and Detection—An Umbrella Review

**DOI:** 10.1002/cre2.70386

**Published:** 2026-06-07

**Authors:** Vini Mehta, Mahati Bhadania, Arpita Singh, Cosimo Galletti, Ankita Mathur

**Affiliations:** ^1^ Faculty of Dentistry University of Ibn al‐Nafis for Medical Sciences Sana'a Yemen; ^2^ Department of Dental Research Cell, Dr. D. Y. Patil Dental College & Hospital Dr. D. Y. Patil Vidyapeeth (Deemed to be University) Pune India; ^3^ Department of Public Health Dentistry, Kalinga Institute of Dental Sciences (KIDS) Kalinga Institute of Industrial Technology (KIIT) Deemed to be University Bhubaneswar India; ^4^ School of Medicine and Surgery Kore University of Enna Enna Italy

**Keywords:** artificial intelligence, cleft lip/congenital, cleft palate/congenital, diagnostic imaging, systematic review

## Abstract

**Purpose:**

Early diagnosis and treatment are essential in managing congenital cleft conditions. Use of artificial intelligence (AI) in routine diagnosis can substantially improve management of chronic conditions; however, in the context of CL/P, evidence remains fragmented. Thus, an umbrella review was planned to explore the applications of AI‐based diagnostic systems in managing orofacial clefts.

**Methods:**

Priori protocol was registered with PROSPERO. Five electronic databases were thoroughly searched (PubMed/MEDLINE, Scopus, Embase, Google Scholar, ScienceDirect), based on the pre‐defined PICO framework. The data extraction form was designed in accordance with the Joanna Briggs Institute (JBI) guidelines and analyzed. Results were presented in the form of tables, supported by narrative summaries. Overlap assessment was conducted to avoid overemphasis and duplication of the overall results. Methodological robustness of the included studies was assessed using AMSTAR 2.0 tool.

**Results:**

Of 395 initially retrieved articles, only three systematic reviews were included for this study. Overlap assessment indicated a high percentage of overlap among studies, with the corrected covered area to be 13.89%. Overall analysis revealed that AI models—DCNN (97% to 96%), RF (99% to 96%), and SVM (94% to 93%) demonstrated high accuracy in diagnosing orofacial clefts. Deep learning models were extensively used in diagnosing and predicting the orofacial cleft with accuracies across models ranging over 90%. Machine learning models also demonstrated good performance in identifying genetic risk. However, the lowest accuracies were demonstrated by the DesNet model (nearly 73% accuracy). Also, methodological robustness indicated a moderate level of confidence, suggesting limitations in the generalizability of the findings.

**Conclusions:**

Findings present the potential of AI models in diagnosing and detecting orofacial clefts. AI models can support the early diagnosis of orofacial clefts; however, this study highlights the need for more comprehensive research to determine how different AI models can improve early diagnosis and treatment outcomes.

**PROSPERO Registration Number:** CRD420251125934.

## 1 Introduction

Orofacial cleft (cleft lip and/or cleft palate: CL/P) is a congenital condition associated with significant functional, developmental, and psychosocial challenges (Baeza‐Pagador et al. 2024; Cleft lip and palate 2017). About 1 in 1000 babies are born with CL/P every year, and the condition is associated with feeding difficulties, increased incidence of ear diseases, and impaired speech and cognitive development (Miller 2011; Smarius et al. 2021; Watkins et al. 2014). Thus, early identification and accurate diagnosis are essential for effective management and timely intervention. With the advancement of technology, the use of Artificial Intelligence (AI) and computational models has demonstrated promising potential in dentistry, as well as in developing personalized treatment plans through AI‐powered tele‐dentistry (Ding et al. 2023).

Recent applications of AI in the dental field have been observed in various domains. For instance, in the field of prosthodontics, the integration of AI‐based care has helped dentists accurately identify dental margins, which is a critical step in the process of dental restorations (Al Hendi et al. 2024). In fact, in the field of surgical intervention, AI‐assisted robots have performed dental implants with more than 90% accuracy (Mehta et al. 2025; Ravipati 2024; Tao et al. 2022). Similarly, in fields such as orthodontics and radiology, AI has shown improvement in diagnostic precision and patient‐centric care (Allareddy et al. 2021; Kazimierczak et al. 2024). A significant number of studies have also concluded that convolutional neural network (CNN) systems have demonstrated high sensitivity and precision in identifying and recognizing dental panoramic radiographs, especially in tooth detection and numbering (Gracea et al. 2025). However, the integration of AI in routine dental care faces many limitations, including issues with data quality used to train AI models, practical implementation challenges, and the proven clinical usefulness (Ghaffari et al. 2024; Huang et al. 2023; Kale et al. 2024).

Nonetheless, with the application of AI‐integrated software, panoramic radiographs or Cone Beam Computed Tomography (CBCT) have shown significant strides in enhancing cleft care and management (Arslan et al. 2024). AI‐facilitated tools not only enhance clinical efficiency but also help reduce time, effort, and costs for patients. They are used for imaging diagnosis (Kuwada et al. 2021), intraoral diagnosis (Santos et al. 2025), surgical intervention (Liu et al. 2019), speech language therapy (Saxon et al. 2020), caregiver education (Harrison et al. 2021), risk prediction (Lim et al. 2021), and risk prediction (Zhang et al. 2018). However, as a relatively new technology, many AI models have not yet been fully validated, particularly in developing countries where resources and data remain limited. The lack of high‐quality training datasets further reduces the generalizability of these models (Nguyen et al. 2026). While existing literature appreciates technology‐assisted perinatal imaging for diagnostic purposes (Bachnas et al. 2024; Lai et al. 2022), the highest level of available literature regarding orofacial clefts has shown fragmented views. The models developed so far show promising results in controlled settings. However, they have not been prospectively tested in different clinical settings, and thus, their applicability cannot be generalized. Therefore, this umbrella review was planned to explore the applications of AI‐based diagnostic systems in managing orofacial clefts. Furthermore, this review will also conduct a critical appraisal of the included studies to demonstrate methodological rigor and fill research gaps, informing future clinical and research applications. This review will fill that gap by summarizing and critically appraising the highest level of available evidence, collating consistent findings, highlighting limitations, and guiding future research and clinical adoption of AI in the diagnosis of CL/P.

## 2 Methods

### 2.1 Protocol Registration

A priori protocol was registered with the International Prospective Register of Systematic Reviews (PROSPERO; registration ID: CRD420251125934). Findings of this review study were reported in accordance with the guidelines of the Joanna Briggs Institute (JBI) (Aromataris et al. 2024) (Table S1).

### 2.2 Focused Review Research Question

A research question was “What is the role of AI‐assisted diagnostic systems (Intervention) in diagnosing CL/P in affected individuals (population) and how accurate are these AI systems?,” aiming to fill the current literature gap in this domain.

### 2.3 Search Databases

Five databases, such as PubMed/MEDLINE, Scopus, Embase, Google Scholar, and ScienceDirect, were used to extract the most relevant research records for this review. A search strategy was developed based on the PICO framework by two independent reviewers to thoroughly explore this subject in depth. Furthermore, Google Scholar was also used only to extract gray literature from the first 50 pages of the obtained results (*n* = 321 pages). As the prompt given generated a large number of results (> 20,000), the search was limited to the first 50 pages only. Reference citation screening was also performed to recover potentially relevant literature. There was no set timeframe for the inclusion of eligible research records; therefore, any eligible study up to August 31, 2025, was considered for screening. The search was conducted across databases from 7th September 2025 to 12th September 2025, by two independent reviewers.

### 2.4 Search Strategy

A comprehensive search strategy was built surrounding the PICO framework: Population (P)‐patient radiographs with CL/P, analyzed by AI‐assisted diagnostic tools (intervention‐ I) compared with conventional methods (comparator‐ C; if reported by included studies) to assess the role of AI in diagnosis and early detection abilities of CL/P congenital anomaly (O). Search strings were developed using Medical Subject Headings [MeSH] key terms with additional fields such as “Title/Abstract or All Fields” with filters such as systematic review (SR) and meta‐analysis (MA), to narrow down the search strategy. Boolean operators, such as “AND,” and “OR,” were extensively used to combine the different search terms wherever needed. A detailed search strategy is listed in Table 1 for PubMed. For other databases, search strategies are listed in Table S2.

**Table 1:** Search strategy used to retrieve relevant evidences.

Database	Search strategy
PubMed	(((((((“artificial intelligence”[All Fields]) OR (“machine intelligence”[All Fields])) OR (“deep learning”[All Fields])) AND (“cleft lip”[MeSH Terms] OR “cleft lip/congenital”[MeSH Terms^a^] OR “cleft lip/diagnostic imaging”[MeSH Terms] OR “cleft lip/diagnosis”[MeSH Terms])) OR (“cleft palate”[MeSH Terms] OR “cleft palate/analysis”[MeSH Terms] OR “cleft palate/congenital”[MeSH Terms] OR “cleft palate/diagnosis”[MeSH Terms] OR “cleft palate/diagnostic imaging”[MeSH Terms])); ((“cleft lip”[MeSH Terms] OR “cleft lip/congenital”[MeSH Terms] OR “cleft lip/diagnostic imaging”[MeSH Terms] OR “cleft lip/diagnosis”[MeSH Terms])) OR (“cleft palate”[MeSH Terms] OR “cleft palate/analysis”[MeSH Terms] OR “cleft palate/congenital”[MeSH Terms] OR “cleft palate/diagnosis”[MeSH Terms] OR “cleft palate/diagnostic imaging”[MeSH Terms])) AND (“artificial intelligence”[MeSH Terms])) AND (“diagnostic imaging”[MeSH Terms]); (((“smart devices”[All Fields]) OR (“artificial intelligence”[All Fields])) AND (“cleft lip”[MeSH Terms] OR “cleft lip/congenital”[MeSH Terms] OR “cleft lip/diagnosis”[MeSH Terms] OR “cleft lip/diagnostic imaging”[MeSH Terms])) OR (“cleft palate”[MeSH Terms] OR “cleft palate/analysis”[MeSH Terms] OR “cleft palate/congenital”[MeSH Terms] OR “cleft palate/diagnosis”[MeSH Terms] OR “cleft palate/diagnostic imaging”[MeSH Terms])

a MeSH: Medical subject headings.

### 2.5 Eligibility Criteria

The retrieved research records were only considered for inclusion if:

a. The study has been a systematic review and/or meta‐analysis (SR/SRMA) in its design.
b. Studies focusing on CL/P radiographs or ultrasound systems where AI‐integrated diagnostic systems were used for identifying this anomaly.
c. Studies were published in peer‐reviewed journals and without any language restrictions (non‐English study articles were systematically translated into English, according to the methodology given by Balk et al. (2012)).

However, all those research studies that did not focus on CL/P were excluded. Furthermore, study designs such as scoping reviews, rapid reviews, and criteria reviews were excluded from the scope of this study. A detailed list of excluded articles is provided in Table S4.

### 2.6 Study Selection

The search results were imported to the Rayyan AI software (Belbasis et al. 2022; Ouzzani et al. 2016) for screening purposes. Two independent reviewers screened the research records using a multiple‐stage screening process. All the potentially eligible studies were further evaluated against the study's eligibility criteria. Shortlisted SR/SRMAs were further screened based on the full‐text of the eligible study articles. Only those studies for which full‐text articles were retrieved were included in the final review. Cohen's kappa for the level of agreement between the independent authors was 0.92. Any conflict raised during the screening process was resolved through mutual discussion and consensus with the third reviewer.

### 2.7 Data Extraction, Data Management, and Data Analysis

A systematic data extraction form was prepared using an MS Excel spreadsheet (version 2402) according to the JBI guidelines. The data were extracted on 12 and 13 August 2025 by two independent reviewers using a standard data extraction form, which includes study ID (authors/year), region, protocol registration, guidelines followed, search duration, databases covered, study design, sample population and size, meta‐analysis information, and ROB tool. Furthermore, intervention characteristics were reported in terms of AI‐model characteristics, including model accuracy, separately from the overall performance of these models. The extracted data was carefully analyzed, and findings were reported in the form of a narrative summary. A quantitative meta‐analysis was not undertaken due to substantial heterogeneity across included systematic reviews in terms of AI model architectures, input datasets (radiographic vs. genetic), imaging modalities (CBCT, panoramic, ultrasound), outcome definitions, and variability in reporting of performance metrics (accuracy, precision, recall, F1‐score), which precluded meaningful statistical pooling.

### 2.8 Assessment of the Overlap of Primary Studies

SRs included in this review were evaluated for overlap among primary studies by constructing a citation matrix (overlap percentage, covered area, corrected covered area), based on the methodologies described by Pieper et al. (2014). Overlap percentage refers to the proportion of the number of studies shared by at least two SRs. Covered area (%) was calculated as the total number of publications (considering only research results related to AI‐integrated biomarker diagnostic methods reported in the included SRs) divided by the product of the total number of studies and the total number of SRs. Corrected covered area (%) was calculated by subtracting the number of unique studies from the total number of publications in each review and dividing the result by the difference between the total study count and the product of the total number of studies and SRs.

This analysis was also performed by two reviewers with good agreement (Cohen's kappa ranging from 0.86 to 0.94). The result was interpreted according to the classification in Table 2.

**Table 2:** Citation matrix interpretation on the basis of the percentage observed.

Interpretation	Overlap %	Covered area %	Corrected covered area %
Mild overlap	< 25	< 10	0–5
Moderate overlap	25–50	11–25	6–10
High overlap	51–75	25–40	11–15
Very high overlap	> 75	> 40	> 16

### 2.9 Outcomes Measured

The primary outcome was to summarize the performance of different AI‐integrated diagnostic systems in detecting and identifying CL/P. Furthermore, secondary outcomes focused on the types of AI models, the algorithms used, the tasks performed, and the limitations of the AI models employed.

### 2.10 Quality Assessment

The included studies were assessed for overall quality of presented evidence using Assessment of Multiple Systematic Reviews 2 (AMSTAR 2) critical appraisal tools (Shea et al. 2017). This tool included eight critical and eight noncritical domains, which evaluate the overall confidence of each SR. Responses were recorded in the form of “Yes,” “Partial Yes,” and “No,” which indicated the overall quality of the included article as “critically low,” “low,” “moderate,” or “high.” Any disagreement was resolved through mutual discussion and with the assistance of a third reviewer (VM). The level of agreement between them was 0.92 for different sections.

## 3 Results

### 3.1 Study Selection

**Figure 1:**
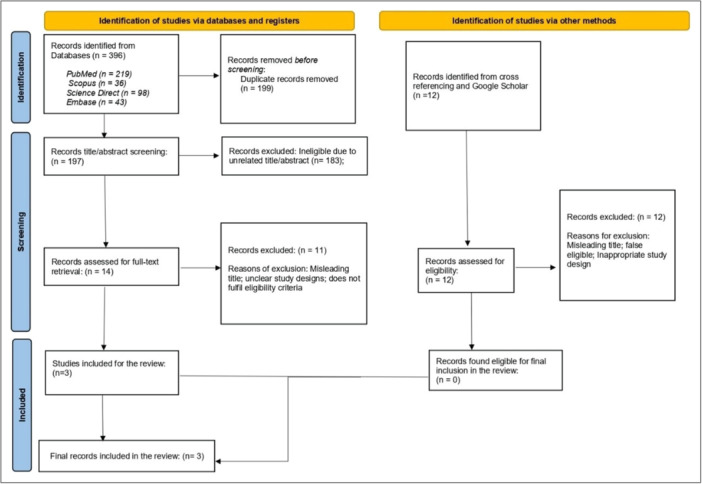
PRISMA flowchart explaining the study selection process.

A total of 396 research records were found across five databases. Of these, 199 duplicates were removed prior to the first screening. The pooled results from these databases were imported into the Rayyan AI software for a multistage screening process. During the first screening, only titles/abstracts were screened, which finally resulted in only 14 articles found potentially eligible for full‐text retrieval and screening. Of 14 research records, only three studies were considered for inclusion in the final review. The following PRISMA flow diagram (Figure 1) illustrates the study selection process and the final articles that are included. A detailed list of the PICO framework of included studies is provided in Table S3.

### 3.2 Study Characteristics

Table 3 illustrates the total of three studies included in this umbrella review. The included studies reported a total of 327 primary studies, of which only 18 studies were related to the diagnosis of CL/P. The relevant primary studies were either retrospective case‐control studies or modeling studies. Of all the included SRs, only one study (Huqh et al. 2022) had registered its protocol with PROSPERO, whereas the other two studies did not mention any protocol registration (Shah et al. 2025; Sivari et al. 2023). Interestingly, all the included studies (*n* = 3) have followed the PRISMA guidelines. Table 3 also represents the geographical landscape of the included studies, which showed that the studies were published mainly from the United States, Malaysia, and Turkey (*n* = 1 each). The included reviews reportedly covered the primary research studies from 2021 to 2023. Major databases, including PubMed, EMBASE, Cochrane, Web of Science, Scopus, and Google Scholar, were utilized by almost all the included studies. None of the included studies (*n* = 3) has reported performing meta‐analysis, and considerable heterogeneity in study design, AI model characteristics, datasets, and reported outcome measures further limited the feasibility of conducting a pooled quantitative synthesis in the present umbrella review. The studies have mentioned using radiographic images (panoramic or CBCT). The overall sample size from the included studies (*n* = 3) was 7415. All the included studies have also compared their accuracy with other conventional methods, such as ultrasound or human radiographic interpretation, depending on the specific objective of each study. All the studies reported outcomes in terms of performance matrices of the AI model used. Furthermore, the risk of bias assessment (RoB) was only reported by two of the three included studies (Huqh et al. 2022; Shah et al. 2025), based on the type of primary study included. A detailed list of the excluded studies is provided in Table S4.

**Table 3:** Study characteristics.

Study ID	Region	Protocol registration	Guideline followed	Language restriction	Database	Included studies	Study designs included	Population included	Overall sample size	Outcome measured	Meta‐analysis (heterogeneity)	Risk of bias tool (ROB)
Huqh et al. (2022)	Malaysia	PROSPERO^a^ [CRD42021270601]	PRISMA^b^	Yes (only English)	PubMed, Scopus, Web of Science	12	Retrospective case‐control	Affected children; adults	6623	Performance matrices	Not performed	JBI^c^ critical appraisal checklist
Sivari et al. (2023)	Turkey	Not mentioned	PRISMA	Yes (only English)	PubMed; Google Scholar	101	Modeling studies	Radiographic images (CBCT^d^, Panoramic)	551	Performance metrics	Not performed	Not performed
Shah et al. (2025)	USA	Not mentioned	PRISMA	Yes (only English)	PubMed, Embase, Web of Science.	25	Modeling studies	Radiographic images (CBCT, Panoramic)	241	Measures of effectiveness or impact	Not performed	PROBAST^e^

^a^ PROSPERO: International prospective register of systematic reviews.

^b^ PRISMA: Preferred reporting items for systematic reviews and meta‐analyses.

^c^ JBI: Joanna Briggs Institute.

^d^ CBCT: Cone beam computed tomography.

^e^ PROBAST: Prediction model Risk of Bias Assessment Tool.

#### 3.2.1 Overlap Assessment

The overlap assessment was performed across included studies (*n* = 3) to assess the uniqueness among studies contributing towards the overall evidence generation. Analysis indicated overlap percentage among primary studies included in the SRs was moderate 27.78% with the uncorrected covered area to be 25.56%. Furthermore, the corrected covered area was found to be 13.89%, indicating high overlap among included studies. Findings for overlap assessment highlight that high overlap may influence the overall findings of this study. A detailed calculation matrix has been provided in Table S5. Furthermore, the absence of meta‐analysis across included studies, coupled with methodological heterogeneity, supports the reliance on a narrative synthesis approach in the present review.

### 3.3 Artificial Intelligence Model Characteristics Used in Diagnosing CL/P

Table 4 illustrates AI models associated with orofacial cleft formations. The comparative table has been expanded to include key methodological descriptors such as dataset type, input modality (e.g., CBCT, panoramic imaging, SNP data), model training approach (supervised/unsupervised), validation characteristics (where reported), and clinical application domains to enhance cross‐study comparability. The analysis showed that out of three studies, only one investigated the accuracy of machine learning algorithms in diagnosing cleft lip and palate (CL/P) (Huqh et al. 2022). The remaining two studies investigated deep learning algorithms in assessing aspects of cleft development during early stages and the diagnostic accuracy of AI‐integrated tools (Shah et al. 2025; Sivari et al. 2023). Analysis revealed that the machine learning models demonstrated diagnostic accuracy of 94.5% (range: 0.92*–*0.95) when random forest or multilayer neural networks were employed, whereas the deep learning model achieved the highest accuracy ranges (0.95–0.96) with DetectNet.

**Table 4:** Artificial intelligence model (with dataset, input modality, and training characteristics).

Study ID	Artificial intelligence model								Comparator given	Overall result
	Model classification (purpose of design)	Model sub‐classification	Algorithm category	Input in model	Model limitation	Model application	Challenges in deploying AI‐based solutions	Opportunities available		
Huqh et al. (2022)	Machine learning (for genetic risk assessment in non‐syndromic CL/P^a^)	SVM^b^, LR^c^, NB^d^, DT^e^, RF^f^, k‐NN^g^	Not explicitly reported (training approach unclear in source SR)	SNPs (genetic dataset input for model training)^i^	Limited scalability and feasibility	Helps in predicting cleft formation during perinatal diagnosis	Applicability cannot be generalized. Tool development is reported to be challenging	Longitudinal studies at multi‐center level	Human intelligence/other diagnostic methods	The combination of these SNPs detected during perinatal screening enables early diagnosis of NSCL + P^j^ with highest accuracy.
		RF								
		ANN^h^								
Sivari et al. (2023)	Deep learning (assessing diagnostic performance and accuracy)	DetectNet	Partially supervised learning	Meta‐analysis (CBCT and panoramic imaging; supervised DL architecture^l^)	Applicability cannot be generalized	Helps in the development of smart diagnostic tools	Quality of training data used to train the model	Geographically diverse dataset trained model are required	Dentists	Highly accurate performance by deep learning model (McNemar's 2 test, *p* < 0.05)
		VGG16^k^								
		3DU‐Net								
Shah et al. (2025)	Deep learning (effectiveness or impact of using an AI‐assisted diagnostic method)	DNN	Supervised and unsupervised learning approaches (hybrid training strategy using radiographic datasets)	Radiographic image datasets (CBCT and panoramic imaging; supervised DL architecture)	Applicability cannot be generalized	High potential to improve cleft care	Quality of dataset; cost of implementation	Validate the efficacy of AI‐driven technologies on a more extensive dataset	Non‐AI based approaches to cleft care	AI has potential to generate highly accurate model if deep learning subset neural networks are used in different forms

^a^ CL/P: Cleft lip and palate.

^b^ SVM: Support vector machine.

^c^ LR: Logistic regression.

^d^ NB: Naive Bayes.

^e^ DT: Decision tree.

^f^ RF: Random forest.

^g^ k‐NN: Kernel neural network.

^h^ ANN: Artificial neural network.

^i^ SNP: Single Nucleotide polymorphisms.

^j^ NSCL + P: Non‐syndromic cleft lip and palate.

^k^ VCG 16: Visual geometry group.

^l^ CBCT: Cone beam computed tomography.

To improve clarity and consistency, model performance metrics across included studies have been standardized and are reported as ranges and/or best‐performing values, depending on data availability from the included systematic reviews. One of the three studies did not clearly mention whether they used supervised or unsupervised learning models to train the developed model on a dataset (Huqh et al. 2022). However, one study by Shah et al. (2025) used both supervised and unsupervised learning methods to train an AI model. The cumulative accuracy remained high for supervised deep learning models (77%–95%) compared to unsupervised ones and machine learning models. Furthermore, while models were trained to perform the function of genetic risk assessment in non‐syndromic CL/P, and to improve diagnostic accuracy. All the studies reported using radiographic images (panoramic and CBCT) as input to train the models. Comparators used in the included studies were either human dentists or those examining human ultrasound. While the included studies (*n* = 3) demonstrated high accuracy in diagnosing CL/P, there were many challenges and limitations observed. One of the major limitations highlighted by all the included studies was the scalability and feasibility of implementing the AI models, as well as the quality of the dataset used for training the models. Among the opportunities, the included studies have mentioned that integrating AI systems into diagnostic tools will not only improve early detection techniques but also leverage smart cleft care methods, especially in developing countries and low‐resource settings. Overall analysis revealed that AI integration into cleft diagnostic systems offers promising results compared to human dentists or radiology examiners. Even if having high reported accuracy, variability in dataset characteristics, model architectures, and validation strategies across studies limits the direct comparability of performance metrics.

### 3.4 Overall Performance of Different AI Models in Diagnosing CL/P

Different AI models perform differently while diagnosing CL/P among the patients, depending upon model objectives. Table 5 illustrates the diagnostic performance of these models in the form of a standardized performance matrix from the findings of non‐duplicated primary studies identified through overlap assessment.

**Table 5:** Performance matrix of different AI models in cleft lip and palate.

Performance matrix*	SVM^a^	KNN^b^	DCNN^c^	ResNet‐50	DenseNet‐169	VGG^4^‐16	RepLKNet	RF^d^	LR^e^	ANN^f^	DT^g^
Accuracy	0.94	0.89	0.96	0.87	0.92	0.81	0.91	0.92	0.95	0.73	0.88
Precision	0.94	0.89	0.96	0.76	0.89	0.70	0.85	0.93	0.91	0.76	0.88
Recall	0.93	0.88	0.97	0.85	0.88	0.95	0.84	0.93	0.90	0.70	0.88
F1‐Score	0.94	0.88	0.96	0.76	0.76	0.78	0.81	0.93	0.90	0.72	0.88

^a^ SVM: Support vector machine.

^b^ k‐NN: Kernel neural network.

^c^ DCNN: Deep convolutional neural network.

^d^ RF: Random forest.

^e^ LR: Logistic regression.

^f^ ANN: Artificial neural network.

^g^ DT: Decision tree.

* All performance metrics are presented as proportions (0–1 scale) derived from non‐duplicated primary studies. Where multiple performance values were reported across studies, representative (best‐reported) values are presented in Table 5, while variability across studies is described in Section Results, 3.

These non‐duplicated studies were identified from overlap assessment SRs. Overall, it was found that DCNN (accuracy range: 0.96; mean = 0.96) and RF (accuracy range: 0.92–0.99; best reported performance: 0.99) outperformed other AI models in diagnosing, predicting, and identifying genetic risks associated with orofacial clefts.

#### 3.4.1 Applications of AI Models in the Diagnosis and Prediction of CL/P

All three included studies have discussed applications of AI models in predicting and diagnosing CL/P. Huqh et al. (2022) reported that MLP models with three hidden layers and 28 perceptrons achieved the highest classification accuracy (92.6%). Furthermore, a study by Sivari et al. (2023) reports that the DetectNet model achieves 95.00% accuracy, making it an important tool for diagnosing and predicting CL/P. Lastly, a study by Shah et al. (2025) reported that when perinatal ultrasound diagnosis was performed, DCNN showed the highest accuracy of 96%, indicating the importance of advanced models in the early diagnosis of CL/P (Table 5).

#### 3.4.2 Applications of AI Models in Identifying Genetic Risks

Only one of the three included studies focused on the implications of using AI models in genetic risk identification. A study by Huqh et al. (2022) reported that AI models, particularly RF (92%) and SVM (94%), improved the detection of variation in methylenetetrahydrofolate reductase and retinol‐binding protein 4, which are crucial in CL/P development. Therefore, early detection of such gene‐level variation using AI models, particularly DenseNet‐169 (accuracy=89%) and ANN (accuracy=73%), can be very helpful in supporting early diagnosis of CL/P, particularly in prenatal conditions (Table 5).

#### 3.4.3 Applications of AI Models in Dental and Sagittal Jaw Relationship

Two of the three studies have noted that Huqh et al. (2022) reported that AI models, particularly LR and WebCeph, enable faster analysis of craniofacial structures with 91% to 95% accuracy in detecting deviations relative to normal structures, compared with conventional methods (Table 5). Overall, this study reports that children with clefts have a smaller angle between SNA and ANB, along with reduced Wits appraisals, indicating a sagittal midface defect. Furthermore, a study by Sivari et al. (2023) highlighted that AI models, particularly VGG and ResNet (accuracy = 70%–81%), improved panoramic images by including “jaw position.”

### 3.5 Assessment of Quality of Included Studies

**Figure 2:**
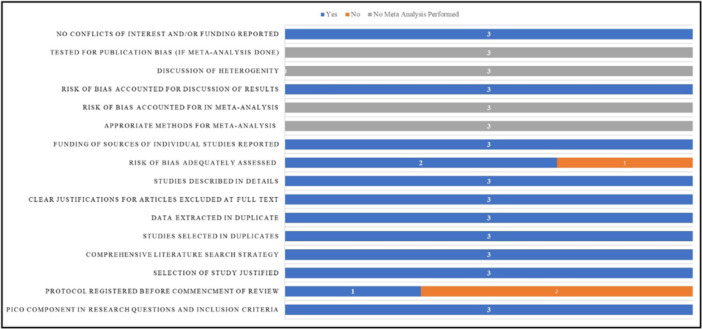
Frequency distribution of included studies across domains of the AMSTAR 2.0 tool.

Figure 2 illustrates the level of confidence in the included studies (*n* = 3) as assessed using the AMSTAR 2.0 tool. The critical appraisal of included studies showed that one of the three included SRs has a high level of confidence (33.33%) in the reported evidence (Huqh et al. 2022) compared to the other two (66.67%) (Shah et al. 2025; Sivari et al. 2023), which showed a moderate level of confidence. This suggests that evidence from high‐quality SR provides more methodologically robust support to overall conclusions compared to the moderate‐confidence reviews. However, it must be interpreted carefully as a smaller number of included studies might potentially influence the overall conclusion. Studies that were listed as flawed are mainly due to “no” responses in a few of the critical domain items, namely, “study did not give information on protocol registration” and “assessment of included studies for risk of bias” in their SRs. This suggests that although a moderate level of confidence was observed in the included studies. Furthermore, the analysis revealed that none of the included studies had conducted a meta‐analysis.

## 4 Discussion

This umbrella review investigated the role of AI in diagnosing and detecting CL/P formation. The overall analysis indicated that AI‐integrated systems have high potential in advancing cleft diagnosis techniques even before birth. The findings also indicated that when diagnostic systems, such as ultrasound, are integrated with AI, they can serve as a genetic risk assessor in the development of CL/P. Overall, deep learning models, DCNN, with RF and SVM‐ machine learning models, showed the highest accuracies compared to others. Notably, only one study showed high confidence in methodological robustness, whereas the other two demonstrated moderate confidence, suggesting the requirement of cautious interpretation of the overall findings in this review.

Only three systematic reviews met the predefined eligibility criteria, primarily due to stringent inclusion criteria (AI‐based diagnostic models with validated outcomes and standardized imaging modalities) and substantial methodological heterogeneity across available studies. This limited evidence base inherently restricts the robustness and generalizability of the findings and reduces the overall certainty of evidence. Therefore, the conclusions drawn from this umbrella review should be interpreted with caution and considered exploratory rather than definitive.

Study characteristics revealed that, although a substantial number of primary studies have been conducted on this subject, systematic pooled evidence synthesis was relatively scarce. Evidence on geographical regions active in this area of research was mostly from developed regions, indicating significant evidence gaps and information asymmetry from different parts of the world, particularly low‐ and middle‐income countries (LMICs). This observation has also been noted by Zambrano et al. (2025), indicating that, as an emerging field, their application remains theoretical and complex, thereby limiting practical implementation.

In spite of having promising diagnostic performance, real‐world clinical applicability remains limited. Integration of AI into routine clinical workflows requires substantial infrastructural support, interoperability with imaging systems, clinician training, and adherence to regulatory frameworks. Cost implications, data privacy concerns, and lack of standardized validation pathways further hinder adoption, particularly in LMIC settings where resource constraints and limited technical expertise pose additional barriers. These translational challenges must be addressed before large‐scale clinical implementation can be achieved.

Model characteristics analysis revealed a stratified picture of not just the models tested but also the tasks they performed. For instance, all the models focused on early diagnosis of CL/P except one, which also focused on genetic risk assessment to identify pathways of CL/P development. Deep learning models demonstrated good performance in diagnosing orofacial clefts. DCNN (deep learning algorithm), followed by RF and SVM (machine learning) models, remain important performers within DL and ML models. This can be attributed to the technical capabilities of DL models, which allow them to process large datasets in a very short duration, where integration of a multi‐layered neural structure further enhances their system. (Soffer et al. 2019) and (Almoammar 2024) presented similar views on the performance capabilities of these systems, suggesting that these models were trained specifically to extract intricate features from raw medical radiographic data. Similarly, studies by Lee and Fujita (2020), Lu et al. (2025), and Yamashita et al. (2018) on DL and medical imaging found that the DCNN model enables superior accuracy in lesion detection or classification compared to a radiologist, suggesting the superiority of these systems and their ability to reduce human errors, thereby improving overall clinical decision‐making systems. Interestingly, evidence reported by Wei et al. (2025) and Xiao et al. (2025) also noted that DL models can not only just perform better but also help in reducing the overall cost of the diagnostic imaging tool, which might increase its usability. On the other hand, ML models, on the other hand, highlighted variable performance. This can be attributed to differences in the algorithmic structure, which is built on multiple neural layers, allowing it to learn independently and handle complex data, such as raw medical image data or sounds, differently than others. This has also been observed by Currie et al. (2019), Kuwada et al. (2023), and Shafi et al. (2020), indicating how algorithmic structure enhances the performance of the diagnostic tools, particularly in chronic conditions such as orofacial clefts. However, it must be noted that comparison between AI models and human experts or conventional diagnostic methods should be interpreted with caution, as included SRs present heterogeneous evidence which limits the ability to present any generalized conclusions regarding their performance and precludes robust quantitative synthesis through meta‐analysis. The absence of meta‐analysis should therefore be interpreted in the context of methodological diversity rather than as a limitation of analytical rigor, as statistical pooling under such heterogeneity could lead to misleading or non‐generalizable estimates.

While all three included studies supported the use of AI‐based models in conjunction with diagnostic imaging tools, they have also highlighted certain limitations, challenges, and opportunities. It was found that all the included SRs have indicated limitations in AI models’ scalability and feasibility. Studies by Diao et al. (2022), Kaboré et al. (2022), and Khan et al. (2025) cumulatively reflected upon AI models' role in diagnostic imaging, it also showed that scalability and feasibility can be a challenge. Studies indicate that a lack of skills in using high‐tech devices due to a lack of proper training has limited its scalability, particularly in LMICs. Additionally, the analysis revealed that the generalizability of the results is a significant challenge, as the study population was specific to a particular geographical location. While limitations reduced the model's efficacy, it also presents significant challenges. One of the prominent challenges identified was the process of tool development, which necessitated its implementation through staff training. This can be attributed to the lack of prior exposure to using such an advanced technology, suggesting it creates a barrier in the smooth adoption and implementation process. Studies by Giebel et al. (2025), Koçak et al. (2025), and Saw and Ng (2022) concluded that medical professionals are not accustomed to using these technologies, suggesting challenges to their smooth adoption and use. Interestingly, one of the studies by Currie et al. (2019) highlighted a “black box fear” among healthcare workers, which refers to challenges in implementing or using the developed models. Our analysis further indicated that the quality of training datasets further compromises the accuracy and precision of the models. Studies by Shah and Gautam (2023) also indicated a lack of standardized data, reducing the quality of imaging‐based diagnosis and the precision of measuring imaging‐based traits. Therefore, such challenges and limitations provide a way forward. The studies indicated that longitudinal studies with multicenter trials are required, with more geographically diverse datasets, which can further enable routes of enhancement. None of the studies specified the average age of the patients included.

### 4.1 Strengths and Limitations

This study aimed to summarize the current state of AI in diagnosing CL/P, drawing on the highest available evidence. The review further stratified AI models used in diagnosing orofacial clefts based on their performance, highlighting which model outperforms the others. Not only this, but our review also classified the accuracy performance of each AI model based on its application and use among others. However, this umbrella review also has certain limitations. Available SRs on this subject were very scant; thus, the overall result has been based on them only. This limited number of included reviews (*n* = 3) further constrains the external validity and generalizability of the findings. Furthermore, studies were based on only a few selected populations and from only a few regional places, suggesting limitations in generalizing overall results from this review. One of the three included studies did not perform a quality assessment of the primary studies, thereby compromising the type of data used. Also, two of the studies have not registered their a priori protocol, indicating the potential for deviation from the proposed study protocol. Moreover, methodological confidence was variable among included studies, which may influence certainty in the overall finding of this study and limit the strength of interpretation among assessed AI models. Additionally, there was observed variability in the comparison as reported by the included SRs; the certainty of the evidence regarding comparative effectiveness between AI models and human experts cannot be conclusively stated. Also, the pooled quantitative estimates (meta‐analysis) could not be performed, primarily due to heterogeneity in AI models, datasets, imaging modalities, validation strategies, and inconsistent reporting of performance metrics across studies, which limits the quantitative strength of the evidence.

## 5 Conclusion

Our study demonstrated the potential of an AI‐assisted diagnostic system to manage orofacial clefts. Current application of AI has shown potential to improve cleft care based on radiographic and dental/facial analysis. However, findings have also revealed challenges and limitations of using AI models in cleft diagnosis and detection. Since having the very limited and heterogeneous evidence base, and the absence of a pooled quantitative synthesis, the certainty of conclusions remains constrained. Authors underscore the importance of more comprehensive research studies for understanding the performance of AI models in comparison to other non‐AI methods or dentists.

## Author Contributions

Conceptualization and design: Vini Mehta and Mahati Bhadania. Methodology: Mahati Bhadania and Arpita Singh. Literature search: Vini Mehta and Mahati Bhadania. Screening and selection: Ankita Mathur and Cosimo Galletti. Data extraction and quality assessment: Mahati Bhadania, Ankita Mathur, and Cosimo Galletti. Data analysis and synthesis: Mahati Bhadania and Arpita Singh. Writing—original draft preparation: Vini Mehta, Mahati Bhadania, and Arpita Singh. Writing—review and editing: Ankita Mathur and Cosimo Galletti. Supervision: Vini Mehta and Ankita Mathur. All authors approved the final manuscript and agree to be accountable for all aspects of the work.

## Funding

The authors have nothing to report.

## Ethics Statement

The authors have nothing to report.

## Consent

The authors have nothing to report.

## Conflicts of Interest

The authors declare no conflicts of interest.

## Supporting information


**Table S1:** PRIOR Checklist.
**Table S2:** Elaborated search strategy.
**Table S3:** PICO elements of included studies.
**Table S4:** List of excluded articles.
**Table S5:** Assessment of Overlap.

## Data Availability

All supporting data are provided in the supporting material accompanying this manuscript.
