# Supplementary material for: Role of Artificial Intelligence in Cleft Lip and/or Cleft Palate in Diagnosis and Detection—An Umbrella Review
Source: Clin Exp Dent Res. 2026 Jun 7;12(3):e70386. doi: 10.1002/cre2.70386 (PMC13242879; doi:10.1002/cre2.70386)
Supplement: Supplementary file 1 — Table S1: PRIOR Checklist. Table S2: Elaborated search strategy. Table S3: PICO elements of included studies. Table S4: List of excluded articles. Table S5: Assessment of Overlap. [file CRE2-12-e70386-s001.docx]

**SUPPLEMENTARY MATERIALS**

**Table S1: PRIOR Checklist**

**(Gates M, Gates A, Pieper D, et al. Reporting guideline for overviews of reviews of healthcare interventions: development of the PRIOR statement. BMJ 2022;378:e070849. doi:10.1136/bmj-2022-070849.)**

| **Section**  Topic | **#** | **Item** | **Location reported** |
| --- | --- | --- | --- |
| **TITLE** | | |  |
| Title | 1 | Identify the report as an overview of reviews. | Page 1 |
| **ABSTRACT** | | |  |
| Abstract | 2 | Provide a comprehensive and accurate summary of the purpose, methods, and results of the overview of reviews. | Page 1 |
| **INTRODUCTION** | | |  |
| Rationale | 3 | Describe the rationale for conducting the overview of reviews in the context of existing knowledge. | Page 1 |
| Objectives | 4 | Provide an explicit statement of the objective(s) or question(s) addressed by the overview of reviews. | Page 2 |
| **METHODS** | | |  |
| Eligibility criteria | 5a | Specify the inclusion and exclusion criteria for the overview of reviews. If supplemental primary studies were included, this should be stated, with a rationale. | Page 2 |
|  | 5b | Specify the definition of ‘systematic review’ as used in the inclusion criteria for the overview of reviews. | Page 3 |
| Information sources | 6 | Specify all databases, registers, websites, organizations, reference lists, and other sources searched or consulted to identify systematic reviews and supplemental primary studies (if included).  Specify the date when each source was last searched or consulted. | Page 3 |
| Search strategy | 7 | Present the full search strategies for all databases, registers and websites, such that they could be reproduced. Describe any search filters and limits applied. | Page 3 |
| Selection process | 8a | Describe the methods used to decide whether a systematic review or supplemental primary study (if included) met the inclusion criteria of the overview of reviews. | Page 3 |
|  | 8b | Describe how overlap in the populations, interventions, comparators, and/or outcomes of systematic reviews was identified and managed during study selection. | Page 3 |
| Data collection process | 9a | Describe the methods used to collect data from reports. | Page 3 |
|  | 9b | If applicable, describe the methods used to identify and manage primary study overlap at the level  of the comparison and outcome during data collection. For each outcome, specify the method used to illustrate and/or quantify the degree of primary study overlap across systematic reviews. | Page 3 |
|  | 9c | If applicable, specify the methods used to manage discrepant data across systematic reviews during data collection. | Page 3 |
| Data items | 10 | List and define all variables and outcomes for which data were sought. Describe any assumptions made and/or measures taken to identify and clarify missing or unclear information. | Not Applicable |
| Risk of bias assessment | 11a | Describe the methods used to *assess* risk of bias or methodological quality of the included systematic reviews. | Page 4 |
|  | 11b | Describe the methods used to *collect* data on (from the systematic reviews) and/or *assess* the risk of bias of the primary studies included in the systematic reviews. Provide a justification for instances where flawed, incomplete, or missing assessments are identified but not re-assessed. | Page 4 |
|  | 11c | Describe the methods used to *assess* the risk of bias of supplemental primary studies (if included). | Not Applicable |
| Synthesis methods | 12a | Describe the methods used to summarize or synthesize results and provide a rationale for the choice(s). | Not Applicable |
|  | 12b | Describe any methods used to explore possible causes of heterogeneity among results. | Not Applicable |
|  | 12c | Describe any sensitivity analyses conducted to assess the robustness of the synthesized results. | Not Applicable |
| Reporting bias assessment | 13 | Describe the methods used to *collect* data on (from the systematic reviews) and/or *assess* the risk of bias due to missing results in a summary or synthesis (arising from reporting biases at the levels of the systematic reviews, primary studies, and supplemental primary studies, if included). | Not Applicable |
| Certainty assessment | 14 | Describe the methods used to *collect* data on (from the systematic reviews) and/or *assess* certainty (or confidence) in the body of evidence for an outcome. | Not Applicable |
| **RESULTS** | | |  |
| Systematic review and supplemental primary study selection | 15a | Describe the results of the search and selection process, including the number of records screened, assessed for eligibility, and included in the overview of reviews, ideally with a flow diagram. | Page 4 |
|  | 15b | Provide a list of studies that might appear to meet the inclusion criteria, but were excluded, with the main reason for exclusion. | Page 5 |
| Characteristics of systematic reviews and supplemental primary studies | 16 | Cite each included systematic review and supplemental primary study (if included) and present its characteristics. | Page 4 |
| Primary study overlap | 17 | Describe the extent of primary study overlap across the included systematic reviews. | Page 5 |
| Risk of bias in systematic reviews, primary studies, and supplemental primary studies | 18a | Present assessments of risk of bias or methodological quality for each included systematic review. | Page 7 |
|  | 18b | Present assessments (*collected* from systematic reviews or *assessed* anew) of the risk of bias of the primary studies included in the systematic reviews. | Not Applicable |
|  | 18c | Present assessments of the risk of bias of supplemental primary studies (if included). | Not Applicable |
| Summary or synthesis of results | 19a | For all outcomes, summarize the evidence from the systematic reviews and supplemental primary studies (if included). If meta-analyses were done, present for each the summary estimate and its precision and measures of statistical heterogeneity. If comparing groups, describe the direction of the effect. | Page 6-7 |
|  | 19b | If meta-analyses were done, present results of all investigations of possible causes of heterogeneity. | Not Applicable |
|  | 19c | If meta-analyses were done, present results of all sensitivity analyses conducted to assess the robustness of synthesized results. | Not Applicable |
| Reporting biases | 20 | Present assessments (*collected* from systematic reviews and/or *assessed* anew) of the risk of bias due to missing primary studies, analyses, or results in a summary or synthesis (arising from reporting biases at the levels of the systematic reviews, primary studies, and supplemental primary  studies, if included) for each summary or synthesis assessed. | Not Applicable |
| Certainty of evidence | 21 | Present assessments (*collected* or *assessed* anew) of certainty (or confidence) in the body of evidence for each outcome. | Not Applicable |
| **DISCUSSION** | | | |
| Discussion | 22a | Summarize the main findings, including any discrepancies in findings across the included systematic reviews and supplemental primary studies (if included). | Page 7 |
|  | 22b | Provide a general interpretation of the results in the context of other evidence. | Page 7 |
|  | 22c | Discuss any limitations of the evidence from systematic reviews, their primary studies, and supplemental primary studies (if included) included in the overview of reviews. Discuss any limitations of the overview of reviews methods used. | Page 8 |
|  | 22d | Discuss implications for practice, policy, and future research (both systematic reviews and primary research). Consider the relevance of the findings to the end users of the overview of reviews, e.g., healthcare providers, policymakers, patients, among others. | Page 8 |
| **OTHER INFORMATION** | | | |
| Registration and protocol | 23a | Provide registration information for the overview of reviews, including register name and registration number, or state that the overview of reviews was not registered. | Page 4 |
|  | 23b | Indicate where the overview of reviews protocol can be accessed, or state that a protocol was not prepared. | Not Applicable |
|  | 23c | Describe and explain any amendments to information provided at registration or in the protocol. Indicate the stage of the overview of reviews at which amendments were made. | Not Applicable |
| Support | 24 | Describe sources of financial or non-financial support for the overview of reviews, and the role of the funders or sponsors in the overview of reviews. | Title Page |
| Competing interests | 25 | Declare any competing interests of the overview of reviews' authors. | Title Page |
| Author information | 26a | Provide contact information for the corresponding author. | Title Page |
|  | 26b | Describe the contributions of individual authors and identify the guarantor of the overview of reviews. | Title Page |
| Availability of data and other materials | 27 | Report which of the following are available, where they can be found, and under which conditions they may be accessed: template data collection forms; data collected from included systematic reviews and supplemental primary studies; analytic code; any other materials used in the overview of reviews. | All supporting data available in Supplementary Sheet |

**Table S2: Elaborated search strategy**

| **Databases** | **Search Strategy** |
| --- | --- |
| Scopus | ( TITLE-ABS-KEY ( artificial intelligence ) OR TITLE-ABS-KEY ( machine learning ) OR TITLE-ABS-KEY ( deep learning ) OR TITLE-ABS-KEY ( intelligent systems ) AND TITLE-ABS-KEY ( cleft palate ) OR TITLE-ABS-KEY ( cleft lip ) OR TITLE-ABS-KEY ( cleft lip palate ) OR TITLE-ABS-KEY ( cleft lip AND palate ) AND TITLE-ABS-KEY ( diagnostic imaging ) OR TITLE-ABS-KEY ( diagnostic accuracy ) OR TITLE-ABS-KEY ( detection ) ) AND ( LIMIT-TO ( DOCTYPE , "re" ) ); ( TITLE-ABS-KEY ( orofacial cleft ) AND TITLE-ABS-KEY ( artificial intelligence ) AND TITLE-ABS-KEY ( diagnosis ) ) AND ( LIMIT-TO ( DOCTYPE , "re" ) ); TITLE-ABS-KEY ( ( ( "cleft lip" OR "cleft palate" OR "orofacial cleft" ) AND ( "artificial intelligence" OR "machine learning" OR "deep learning" ) ) ) AND ( LIMIT-TO ( DOCTYPE , "re" ) ) |
| Embase | ('cleft palate'/exp OR 'cleft palate' OR 'cleft lip':ti,ab,kw OR 'cleft lip with':ti,ab,kw OR 'without cleft palate':ti,ab,kw) AND 'artificial intelligence':ab,ti AND 'diagnostic accuracy':ab,ti; ('artificial intelligence' OR 'smart device' OR 'machine learning' OR 'deep learning' OR 'artificial neural network') AND ('orofacial cleft' OR 'cleft palate' OR 'cleft lip') AND ('diagnosis' OR 'early diagnosis'); ('artificial intelligence' OR 'smart device' OR 'machine learning' OR 'deep learning' OR 'artificial neural network') AND ('orofacial cleft' OR 'cleft palate' OR 'cleft lip') |
| Science Direct | orofacial cleft AND "artificial intelligence" AND "diagnostic accuracy";  artificial intelligence OR machine intelligence AND "cleft lip OR cleft palate" AND "diagnostic accuracy"; ('smart device' OR 'ultrasound') AND 'artificial intelligence' AND 'cleft palate' OR 'cleft lip with or without cleft palate';   "smart equipment OR intelligence devices OR artificial intelligence" AND "cleft lip OR cleft palate OR orofacial cleft" AND "diagnostic accuracy" |

**Table S3: PICO elements of included studies**

| **Study ID** | **Population (P)** | **Intervention (I)** | **Comparator (C)** | **Outcome (O)** |
| --- | --- | --- | --- | --- |
| Huqh et al. 2022 | SNP^1^ | AI^2^ Model- ML^3^ | Human intelligence/other diagnostic methods which does not involve AI models | Genetic sequence identification and early detection of orofacial cleft |
| Sivari et al. 2023 | Radiographic image datasets (Panoramic, CBCT^4^) | AI Model-DL^5^ | Dentists | Cleft diagnosis and detection |
| Shah et al. 2025 | Radiographic image datasets (Panoramic, CBCT) | AI Model-DL | Non-AI based approaches to cleft care | Cleft diagnosis and detection |

1: SNP: Single nucleotide polymorphism; 2: AI: Artificial intelligence; 3: ML: Machine learning; 4: CBCT: Cone beam computed tomography; 5: DL: Deep learning

**Table S4:** **List of excluded articles**

| **Study Title** | **Author and Year** | **Reason for exclusion** |
| --- | --- | --- |
| Classification, Epidemiology, and Genetics of Orofacial Clefts | Watkins et al. 2014 | This article does not directly focus on the orofacial cleft diagnosis |
| Application of artificial intelligence in treating patients with cleft and craniofacial anomalies | Elnagar et al. 2022 | This article does not directly focus on the orofacial cleft diagnosis |
| The Contemporary Management of Cleft Lip and Palate and the Role of Artificial Intelligence: A Review | Marya et al. 2022 | This article does not directly focus on the orofacial cleft diagnosis |
| [Progress in prenatal ultrasound screening of fetal cleft lip and palate and application of artificial intelligence](https://chaosheng.cma-cmc.com.cn/EN/PDF/10.3877/cma.j.issn.1672-6448.2022.11.022) | Chen et al. 2022 | This article does not meet the study design inclusion criteria (as it is not a systematic review) |
| 3D surface imaging technology for objective automated assessment of facial interventions: A systematic review | Nguyen et al. 2022 | This article does not meet the intervention inclusion criteria of PICO framework |
| Evolving the Era of 5D Ultrasound? A Systematic Literature Review on the Applications for Artificial Intelligence Ultrasound Imaging in Obstetrics and Gynecology | Jost et al. 2023 | This article does not meet the outcome of interest from the PICO framework |
| Artificial Intelligence Applications in Orthodontics | Miranda et al. 2023 | This article does not meet the study design inclusion criteria (as it is not a systematic review) and did not fulfill the eligibility criteria |
| Maternal stress as a risk factor for non-syndromic orofacial clefts: Systematic review and *meta*-analysis | AlSharif et al. 2023 | This article does not directly focus on the orofacial cleft diagnosis |
| Diagnostic Methods for the Prenatal Detection of Cleft Lip and Palate: A Systematic Review | Baeza-Pagador et al. 2024 | This article does not meet the intervention criteria of PICO framework |
| Artificial Intelligence Used for Diagnosis in Facial Deformities: A Systematic Review | Ravelo et al. 2024 | This article does not directly focus on the orofacial cleft diagnosis |
| AI and early diagnostics: mapping fetal facial expressions through development, evolution, and 4D ultrasound | Andonotopo et al. 2025 | This article does not meet the intervention inclusion criteria of PICO framework |

**Table S5: Assessment of Overlap**

| **Primary studies included** | **Huqh et al. 2022** | **Sivari et al. 2023** | **Shah and Yoon et al. 2025** |
| --- | --- | --- | --- |
| Wang et al. 2021 | 1 | 1 | 1 |
| Xu et al. 2017 | 1 |  | 1 |
| Machado et al. 2020 | 1 |  |  |
| Shafi et al. 2020 | 1 |  |  |
| Kuwada et al. 2023 (a) | 1 |  | 1 |
| Sayadi et al. 2022 |  |  | 1 |
| Schnabel et al. 2023 |  |  | 1 |
| Miranda et al., 2023 |  |  | 1 |
| Kuwada et al., 2023 (b) |  |  | 1 |
| Zhang et al. 2018 | 1 |  |  |
| Alam et al., 2021 | 1 |  | 1 |
| Kuwada et al., 2021 | 1 | 1 | 1 |
| Zhang et al., 2022 | 1 |  | 1 |
| Alamand and Alfawzan, 2020 | 1 |  |  |
| Golabbakhsh et al., 2017 | 1 |  |  |
| Wang et al. 2019 | 1 |  |  |
| Orozco-Arroyave et al., 2016 | 1 |  |  |
| Shafi et al., 2020 | 1 |  |  |
| Number of included primary studies (including double counting) SR/SRMAs (N)= 23  Number of columns (number of reviews) (C) = 3  Number of rows (number of index publications) (R) = 18  Total number of overlapping studies (n) = 05  Overlap % (n/R*100) = (05/18) * 100 = 27.78%  CA % [(N/C*R) *100] = (23/5*18) * 100 = 25.56%  CCA % [((N-R)/(C*R-R)) * 100] = 5/36* 100 = 13.89% | | | |

**References**

1. Huqh MZU, Abdullah JY, Wong LS, Jamayet NB, Alam MK, Rashid QF, et al. Clinical Applications of Artificial Intelligence and Machine Learning in Children with Cleft Lip and Palate—A Systematic Review. IJERPH. 2022 Aug 31;19(17):10860.

2. Sivari E, Senirkentli GB, Bostanci E, Guzel MS, Acici K, Asuroglu T. Deep Learning in Diagnosis of Dental Anomalies and Diseases: A Systematic Review. Diagnostics. 2023 Jul 27;13(15):2512.

3. Shah J, Yoon J, Lowe K, Ko J, Oberoi S. Efficacy of artificial intelligence in cleft care: A systematic review. Seminars in Orthodontics. 2025 Mar;S1073874625000295.
